# Causal role of immune cells in thyroid cancer: a bidirectional Mendelian randomization study

**DOI:** 10.3389/fimmu.2024.1425873

**Published:** 2024-06-17

**Authors:** Xianliu Fang, Xiaoxiao Huang, Jianhua Lu, Danke Su

**Affiliations:** ^1^ Department of Medical Imaging Center, Guangxi Medical University Cancer Hospital, Nanning, Guangxi, China; ^2^ Rehabilitation Medicine Department, The First Affiliated Hospital of Guangxi Medical University, Nanning, Guangxi, China

**Keywords:** immunophenotypes, causal inference, Mendelian randomization study, thyroid cancer, immunotherapy

## Abstract

**Background:**

The immune system plays an important role in the development and treatment of thyroid cancer(THCA).However, the correlation between immune cells and THCA has not been systematically studied.

**Methods:**

This study used a two-sample Mendelian randomization (MR) study to determine the causal relationship between immune cell characteristics and THCA. Based on a large sample of publicly available genetic data, we explored the causal relationship between 731 immune cell characteristics and THCA risk. The 731 immunophenotypes were divided into 7 groups, including B cell panel(n=190),cDC panel(n=64),Maturation stages of T cell panel(n=79),Monocyte panel(n=43),Myeloid cell panel(n=64),TBNK panel(n=124),and Treg panel(n=167). The sensitivity of the results was analyzed, and heterogeneity and horizontal pleiotropy were excluded.

**Results:**

After FDR correction, the effect of immunophenotype on THCA was not statistically significant. It is worth mentioning, however, that there are some unadjusted low P-values phenotypes. The odds ratio (OR) of CD62L on monocyte on THCA risk was estimated to be 0.953 (95% CI=0.930~0.976, *P*=1.005×10^−4^),and which was estimated to be 0.975(95% CI=0.961–0.989, *P*=7.984×10^−4^) for Resting Treg%CD4 on THCA risk. Furthermore, THCA was associated with a reduced risk of 5 immunophenotype:CD25 on CD39+ CD4 on Treg (OR=0.871, 95% CI=0.812~0.935, *P*=1.274×10^−4^), activated Treg AC (OR=0.884, 95% CI=0.820~0.953, *P*=0.001), activated & resting Treg % CD4 Treg (OR=0.872, 95%CI=0.811~0.937,*P*=2.109×10^−4^),CD28- CD25++ CD8br AC(OR=0.867,95% CI=0.809~0.930,*P*=6.09×10^−5^),CD28-CD127-CD25++CD8brAC(OR=0.875,95%CI=0.814~0.942,*P*=3.619×10^−4^).THCA was associated with an increased risk of Secreting Treg % CD4 Treg (OR=1.143, 95% CI=1.064~1.229, *P*=2.779×10^−4^) and CD19 on IgD+ CD24+ (OR=1.118, 95% CI=1.041~1.120, *P*=0.002).

**Conclusions:**

These findings suggest the causal associations between immune cells and THCA by genetic means. Our results may have the potential to provide guidance for future clinical research.

## 1 Introduction

Thyroid cancer (THCA) is the most common malignancy of the endocrine system. The incidence of THCA has been increasing over the past few decades (1). According to the GLOBOCAN 2020 Cancer Incidence and Mortality database of the World Health Organization’s International Agency for Research on Cancer, thyroid cancer ranks ninth in the global incidence of cancer (2, 3). Thyroid cancer has become the most common malignancy among adolescents and adults aged 16–33 years (4). Although most patients with THCA are cured by 131I or surgery, a small percentage die from metastasis or recurrence (5). One study showed that 28% of THCA patients relapsed and 9% of THCA patients died (6). Part of the reason is that in the process of tumor development, the interaction and collective action between the tumor and the surrounding immune microenvironment promote immune tolerance, and then develop into immune escape, resulting in the inability of the body to completely clear the tumor. In recent years, important progress has been made in the treatment of tumor immune-related regulation (7, 8). Studies have shown that, the immune system plays a key role in the occurrence and development of tumor. Tumor cells are recognized and eliminated by various immune cells in the body (9). Mantovani A et al. found that both innate and acquired immune responses can promote cancer initiation and tumor growth, or have anticancer effects (10). Previous studies have found that macrophage-targeted therapy can be used for invasive THCA, especially for THCA with high tumor-associated macrophage (TAM) content. Despite immunotherapy, including tumor vaccine therapy, immune checkpoints Inhibitor therapy, adoptive immune cell therapy, monoclonal antibody therapy and immunoregulatory cell targeting therapy have been widely used in the clinical treatment of thyroid cancer (8), but the correlation between immune cells and THCA has not been systematically studied.

Recently, Mendelian randomization (MR) studies based on whole genome sequencing data have been an effective and powerful statistical method to uncover causality by using genetic variation as an instrumental variable (IVs) (11). MR analysis makes use of the fixed nature and heredity of genes, that is, when meiosis gametes are formed, alleles of parents are randomly assigned to offspring, and the relationship between genes and outcome is not interfered by common confounding factors such as postnatal environment, behavioral habits, and social economy, so the causal relationship derived from this is reasonable (12).

In this study, we performed a comprehensive two-sample MR analysis to determine the causal association between immunophenotypes and THCA.

## 2 Materials and methods

### 2.1 Study design

Through a two-sample MR Analysis, we analyzed the causal relationship between 731 immune cell characteristics (7 groups) and THCA. MR analysis uses genetic variation as a working variable to estimate the causal relationship between the exposure factor of interest and the outcome of concern. Therefore, the effective instrumental variables (IVs) in MR Analysis must satisfy three key assumptions:(1) The association hypothesis: genetic variation needs to have a strong correlation with exposure factors, and if weak IVs are used, the results are prone to bias; (2) Independence hypothesis: genetic variation cannot be associated with possible confounders; (3) Exclusivity hypothesis: genetic variation can only affect outcomes through exposure, but not through other factors. Data for analysis included in our study were obtained with the approval of the relevant institutional review boards, and study participants provided informed consent.

### 2.2 Data sources

The genetic variation of each immune trait was obtained from the GWAS catalog (registration numbers GCST0001391 to GCST0002121) (13), which is the largest GWAS published so far on peripheral blood immune phenotypes. The total of 731 immunophenotypes were divided into 7 groups, including B cell panel(n=190),cDC panel(n=64),Maturation stages of T cell panel(n=79),Monocyte panel(n=43),Myeloid cell panel(n=64),TBNK panel(n=124),and Treg panel(n=167).

The initial immune characteristics of GWAS were analyzed using data from 3757 Europeans (57% female) without overlapping cohorts. About 22 million single nucleotide polymorphisms(SNPs) genotyped using high-density arrays were estimated using a reference panel based on Sardinian sequences and tested the correlation after adjusting for covariates (i.e.sex, age) (14). GWAS summary statistics for THCA were obtained from a GWAS, which was downloaded at FinnGen consortiums, including 491,974 European individuals (N_case_ = 1,054, N_control_ = 490,920), with approximately 24.2 million variants analyzed after quality control and imputation.

### 2.3 Selection of instrumental variables

In this study, IVs were selected according to the following selection criteria:(1)SNPs strongly associated with exposure factors were selected (*p*<1×10^−5^); (2)Linkage disequilibrium(LD) was removed using r^2^ <0.1 within 500 KB distance; (3)Instrumental variable correlation assessment: select F>10 to avoid bias caused by weak tools; (4) When palindromic SNPs exist, the allele frequency information is used to infer the front chain allele.

### 2.4 Statistical analysis

We used the following statistical methods to make causal inference of MR Analysis to evaluate the causal relationship between 731 immunophenotypes and THCA:(1)Inverse variance weighting (IVW) (15) was used as the main analysis; (2)Cochran’s Q statistic and MR pleiotropy residual sum and outlier (MR-PRESSO) (16) were used to analyze the heterogeneity of SNPs. (3)MR-Egger (17) and Leave-one-out method were used to analyze the sensitivity of the results. (4) The significant association between immunophenotype and THCA was corrected for false discovery rate (FDR) to control for the proportion of false positives in multiple tests. In addition, according to the IVs generated by THCA GWAS statistical data, reverse MR analysis was conducted to determine whether there was a causal relationship between THCA and immune traits. *P*<0.05 indicates the potential causal relationship in MR analysis, which is statistically significant. All statistical analyses were conducted using the “Two-Sample MR” package in R 4.3.1 software (http://www.Rproject.org).

## 3 Results

### 3.1 Exploration of the causal effect of THCA onset on immunophenotypes

**Figure 1:**
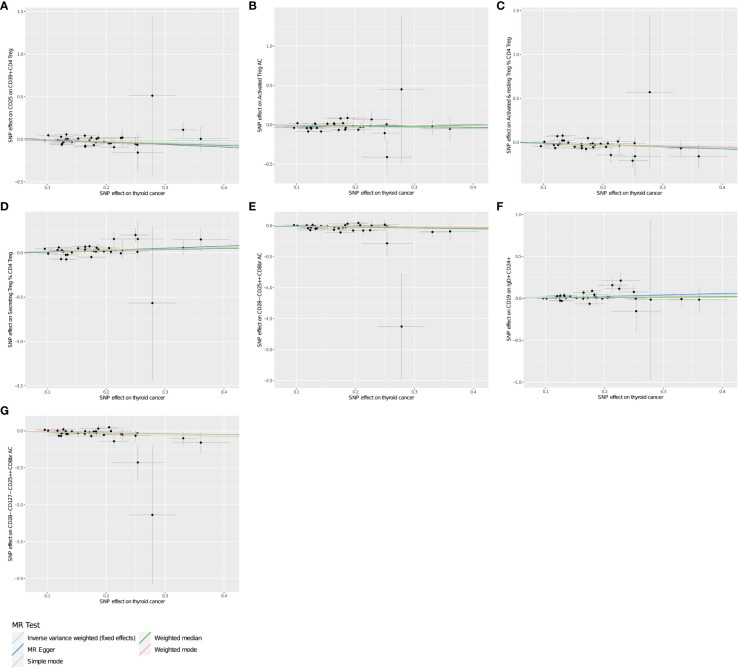
**(A–G)** Scatter plots show the MR effect of THCA onset on immunophenotypes in different MR methods.

**Figure 2:**
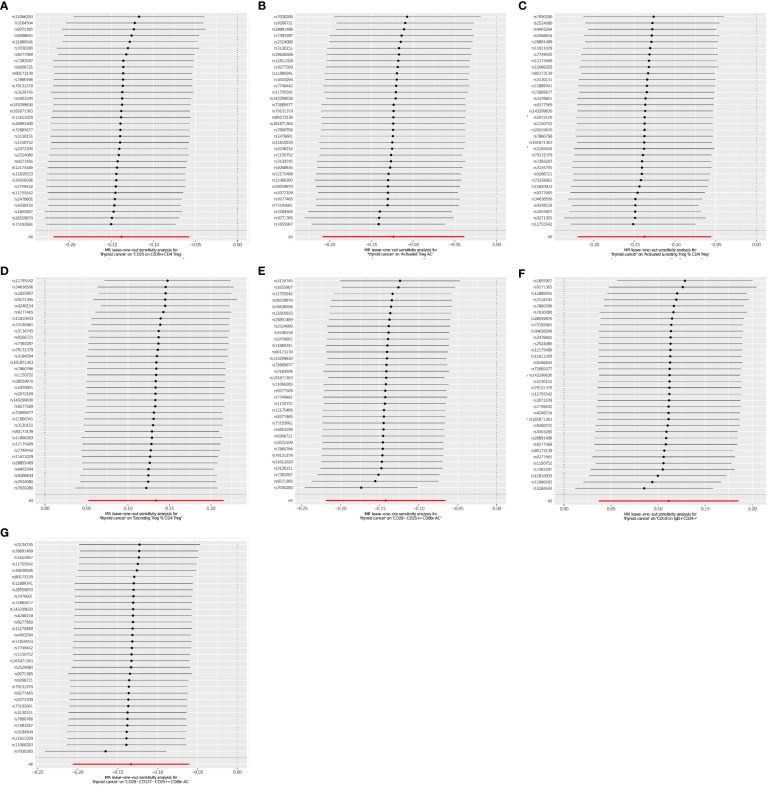
**(A–G)** Leave-one-out plots for the causal effect of THCA onset on immunophenotypes.

**Figure 3:**
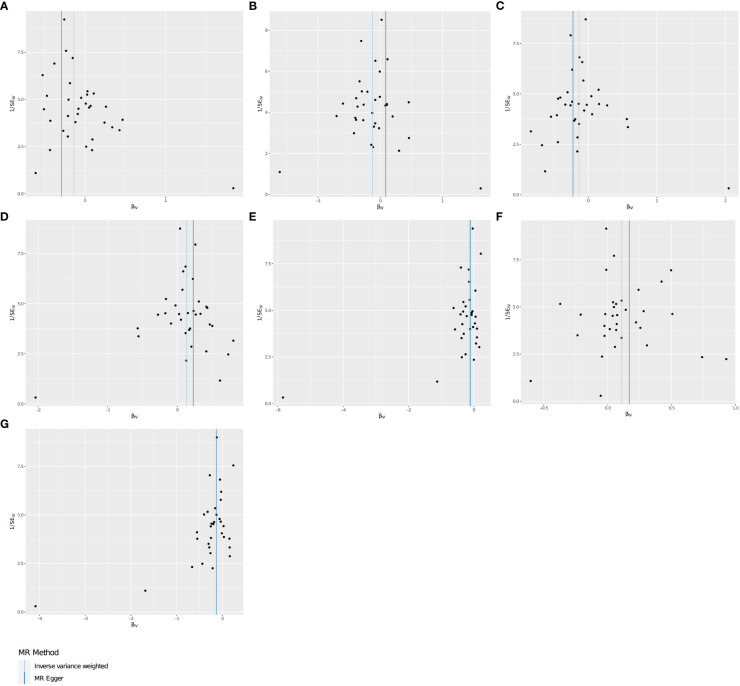
**(A–G)** Funnel plots for the causal effect of THCA onset on immunophenotypes.

Using two-sample MR analysis to explore the causal effects of THCA onset on immunophenotypes. After a variety of tests and adjustments using the FDR method, 7 immune traits were identified with a difference of 0.05, including 6 in the Treg group and 1 in the B cell group. We found that THCA onset could decrease the level of CD25 on CD39+ CD4 on Treg (OR=0.871, 95% CI=0.812~0.935, *P*=1.274×10^−4^, *P*FDR= 0.01, **Table 1**). Activated Treg AC were decreased in THCA patients (OR=0.884, 95% CI=0.820~0.953, *P*=0.001, *P*FDR =0.03, **Table 1**). Activated & resting Treg % CD4 Treg was also found to be decreased (OR=0.872, 95% CI=0.811~0.937,*P*=2.109×10^−4^, *P*FDR = 0.013, **Table 1**). Similar associations were found for CD28- CD25++ CD8br AC (OR=0.867, 95% CI=0.809~0.930, *P*=6.09×10^−5^, *P*FDR = 0.006, **Table 1**), and CD28- CD127- CD25++ CD8br AC (OR=0.875, 95% CI=0.814~0.942,*P*=3.619×10^−4^, *P*FDR = 0.017, **Table 1**). By contrary, We found that THCA onset could increase the level of Secreting Treg % CD4 Treg (OR=1.143, 95% CI=1.064~1.229, *P*=2.779×10^−4^, *P*FDR=0.016, **Table 1**), and CD19 on IgD+ CD24+ (OR=1.118, 95% CI=1.041~1.120, *P*=0.002, *P*FDR = 0.048, **Table 1**). The MR effect of THCA onset on immunophenotypes in different MR method was showed in **Figure 1**. Visual inspection of funnel plots (**Figure 2**) and leave-one-out plots (**Figure 3**) did not reveal any obvious directional pleiotropy.

**Table 1:** MR estimates for the causal effect of THCA onset on immunophenotypes.

traits (outcome)	MR method	No. of SNP	F-statistic	OR	95% CI	*P*-value
CD25 on CD39+ CD4 Treg	IVW	34	63.4	0.871	0.812~0.935,	1.274×10^−4^
	MR-Egger	34	63.4	0.747	0.547–1.019	0.075
	Weighted median	34	63.4	0.840	0.751–0.938	0.002
	Weighted mode	34	63.4	0.825	0.709–0.959	0.017
	Simple mode	34	63.4	0.962	0.769–1.202	0.733
Activated Treg AC	IVW	34	63.4	0.884	0.820–0.953	0.001
	MR-Egger	34	63.4	1.092	0.794–1.502	0.591
	Weighted median	34	63.4	0.923	0.821–1.039	0.184
	Weighted mode	34	63.4	0.972	0.804–1.174	0.767
	Simple mode	34	63.4	0.905	0.721–1.136	0.395
Activated & resting Treg % CD4 Treg	IVW	34	63.4	0.8716	0.811–0.937	2.109×10^−4^
	MR-Egger	34	63.4	0.800	0.585–1.093	0.170
	Weighted median	34	63.4	0.876	0.784–0.979	0.019
	Weighted mode	34	63.4	0.874	0.755–1.011	0.079
	Simple mode	34	63.4	0.850	0.707–1.022	0.093
Secreting Treg % CD4 Treg	IVW	34	63.4	1.143	1.064–1.229	2.779×10^−4^
	MR-Egger	34	63.4	1.256	0.921–1.714	0.160
	Weighted median	34	63.4	1.126	1.010–1.256	0.033
	Weighted mode	34	63.4	1.137	0.985–1.313	0.090
	Simple mode	34	63.4	1.168	0.975–1.400	0.101
CD28- CD25++ CD8br AC	IVW	33	63.6	0.867	0.809–0.930	6.09×10^−5^
	MR-Egger	33	63.6	0.899	0.670–1.205	0.480
	Weighted median	33	63.6	0.885	0.796–0.983	0.022
	Weighted mode	33	63.6	0.925	0.772–1.110	0.409
	Simple mode	33	63.6	0.950	0.774–1.167	0.630
CD19 on IgD+ CD24+	IVW	34	63.4	1.118	1.041–1.200	0.002
	MR-Egger	34	63.4	1.184	0.887–1.583	0.261
	Weighted median	34	63.4	1.054	0.950–1.169	0.324
	Weighted mode	34	63.4	1.030	0.890–1.191	0.698
	Simple mode	34	63.4	1.037	0.867–1.240	0.691
CD28- CD127- CD25++ CD8br AC	IVW	33	63.6	0.875	0.814–0.942	3.619×10^−4^
	MR-Egger	33	63.6	0.876	0.665–1.155	0.355
	Weighted median	33	63.6	0.883	0.794–0.983	0.023
	Weighted mode	33	63.6	0.887	0.759–1.037	0.141
	Simple mode	33	63.6	0.829	0.694–0.990	0.047

MR, Mendelian randomization; SNP, single nucleotide polymorphism; OR, odds ratio; CI, confidence interval; IVW, inverse variance weighted.

### 3.2 Exploration of the causal effect of immunophenotypes on THCA

**Figure 4:**
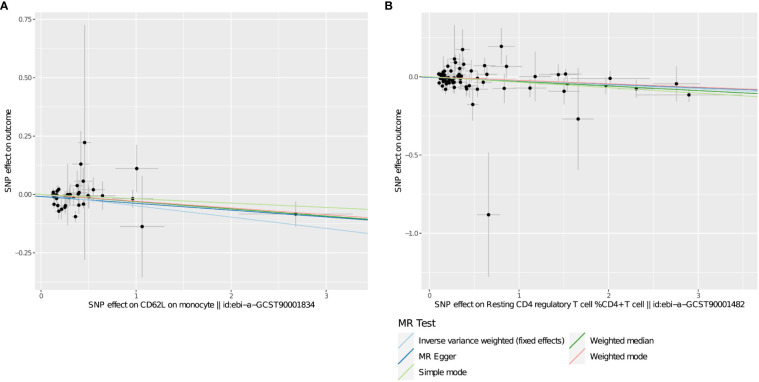
**(A, B)** Scatter plots show the MR effect of immunophenotypes on THCA in different MR methods.

**Figure 5:**
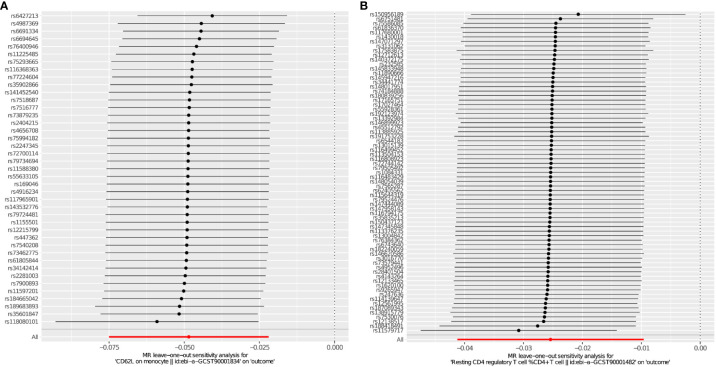
**(A, B)** Leave-one-out plots for the causal effect of immunophenotypes on THCA.

**Figure 6:**
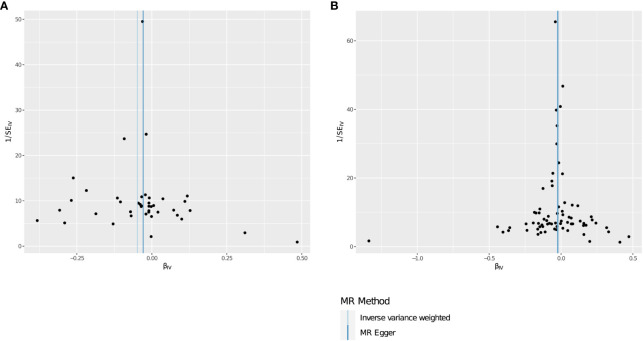
**(A, B)** Funnel plots for the causal effect of immunophenotypes on THCA.

After multiple experimental adjustments based on the FDR method, no immune characteristics at a significance of 0.05 were found. At a significance of 0.20, we detected two immune traits as protective effects against THCA: CD62L on monocyte(cDC panel),with the odds ratio(OR) being estimated to be 0.953 (95% CI=0.930~0.976, *P*=1.005×10^−4^, PFDR=0.073, **Table 2**), and Resting Treg%CD4 (Treg panel),with the odds ratio(OR) being estimated to be 0.975(95% CI=0.961–0.989,*P*=7.984×10^−4^, *P*FDR=0.195, **Table 2**).The MR effect of each exposure on THCA in different MR method was showed in **Figure 4**. Visual inspection of funnel plots (**Figure 5**) and leave-one-out plots (**Figure 6**) did not reveal any obvious directional pleiotropy.

**Table 2:** MR estimates for the causal effect of immunophenotypes on THCA.

traits (exposure)	MR method	No. of SNP	F-statistic	OR	95% CI	*P*-value
CD62L on monocyte	IVW	41	31.3	0.953	0.930~0.976	1.005×10^−4^
	MR-Egger	41	31.3	0.971	0.935–1.009	0.137
	Weighted median	41	31.3	0.970	0.930–1.010	0.143
	Weighted mode	41	31.3	0.972	0.938–1.006	0.112
	Simple mode	41	31.3	0.982	0.918–1.050	0.591
Resting Treg %CD4	IVW	71	35.9	0.975	0.961–0.989	7.984×10^−4^
	MR-Egger	71	35.9	0.978	0.959–0.998	0.031
	Weighted median	71	35.9	0.971	0.950–0.993	0.011
	Weighted mode	71	35.9	0.978	0.961–0.996	0.018
	Simple mode	71	35.9	0.966	0.925–1.009	0.127

MR, Mendelian randomization; SNP, single nucleotide polymorphism; OR, odds ratio; CI, confidence interval; IVW, inverse variance weighted.

## 4 Discussion

The relationship between the immune system and THCA has always been a research hotspot. THCA is a malignant tumor that occurs in thyroid tissue, and the immune system plays an important role in the development and treatment of THCA. In THCA, immune cell interactions or interactions with cancer cells can exert anti-tumor and pro-tumor functions. In addition, various soluble factors released by immune cells (such as chemokines, cytokines, angiogenic factors, etc.) can also mediate the pro-tumor and anti-tumor effects of immune cells in cancer.

Galdiero et al. proposed that the immune system may be involved in the occurrence of THCA (18). Previous meta-analyses and systematic reviews of observational studies have also demonstrated an association between THCA and certain immune factors (e.g., neutrophils, Natural killer T cells, γδ T cells and innate lymphoid cells) (9, 19). However, observational studies may introduce confounding factors, and they cannot always differentiate between symptoms and etiology. Using genetically predicted causal effects of immunophenotypes on THCA, our results do not align with the previous findings.

It is well known that inflammation helps tumor development (20), and the immune system may prevent tumor growth by eliminating infections in a timely manner. Willimsky G et al. found that the immune system seems to have little effect on the onset of tumors, with some parts of the immune system showing a clear protective effect (21). Similarly, in our results, both CD62L on monocyte and Resting Treg %CD4 had protective effects on THCA,and no immune factors were found to contribute to the onset of THCA. CD62L is a cell adhesion molecule and type I transmembrane glycoprotein expressed on most circulating white blood cells. It is widely described as a bound/rolling receptor that plays a role in regulating monocyte protrusion during transendothelial migration (TEM). In adult humans, monocytes account for 2–8% of all circulating white blood cells and classic monocytes express high levels of L-selectin, i.e. CD62L on monocyte. During TEM of monocyte, the detachment of CD62L domains is crucial for establishing pre- and post cell polarity and achieving chemotaxis towards the site of injury (22), which can exert anti-tumor immune effects. The anti-tumor ability of CD4+ T cells has been widely demonstrated in mouse models and humans (23). By activating the immune system, CD4+ T cells can enhance the recognition and elimination of tumor cells, thereby inhibiting tumor growth and spread. Our results show that both CD62L on monocyte and Resting Treg %CD4 have a protective effect on THCA. Enhancing the activity of CD62L on monocyte and resting Treg%CD4 cells in tumor immunotherapy is expected to improve the therapeutic effect and may become an important part of future tumor immunotherapy strategies.

Jena D et al. found that the type of immune response produced by THCA was associated with disease severity (24). Many studies have proved that tumors have strong immunosuppressive ability. Regulatory T cells (Tregs) are a subset of T cells that inhibit the function of many immune cells. Tregs usually accumulate in cancer foci, draining lymph nodes, and peripheral blood (25–27). It is reported that the poor prognosis of many cancers is related to the increase of Tregs, such as breast cancer, ovarian cancer and lymphoma (28–30), and the invasiveness of THCA is related to the increase of Tregs. The analysis of specific lymphocyte indicates that Tregs are consistently present in THCA lesions and peripheral blood, and their increase is correlated with the severity of the disease (23). Our results show that Secreting Treg % CD4 Tregs are significantly elevated in THCA, substantiating previous studies at the genetic level. Although further research is needed to clarify the exact mechanisms of action of Tregs in THCA, the clear elevation of Secreting Treg % CD4 Treg in THCA provides new avenues for future diagnostics and therapeutics.

Regulatory B cells (Bregs) promote tumor immune escape by secreting cytokines such as IL-10, TGF-β1, IL-35, PD-L1 and so on. Recently, it has been found that PD-1+Breg is abundant in thyroid cancer tissue and peripheral blood, and inhibits T cell viability and proliferation in an IL-10-independent manner (31). CD19 molecule is a differentiation antigen on the surface of Breg and one of the characteristic markers on the cell membrane. CD19 can participate in the activation and proliferation of Breg, and is widely expressed in tumors related to the B lymphatic system, but not in plasma cells, T cells, and other tissues. It does not disappear or decay during Breg activation (32). In this study, we performed MR Analysis based on recently published GWAS data, showing that CD19 on IgD+ CD24+ is significantly elevated in Thachik supports previous studies.

Additionally, it was noteworthy that the presence of THCA is related to the reduction of the level of 5 immunophenotypes in Treg group, including CD25 on CD39+ CD4 Treg, Activated Treg AC, Activated & resting Treg % CD4 Treg, CD28- CD25++ CD8br AC, and CD28- CD127- CD25++ CD8br AC. Cancer can stimulate the body’s specific immunity against tumors. However, due to the highly immunosuppressive nature of the tumor microenvironment and the suppression of tumor-associated antigen-specific T cells, the body is unable to eliminate cancer cells. This immunosuppressive microenvironment promotes the growth of cancer cells, leading to tumor recurrence and poor prognosis.

This study conducted a two sample MR analysis on the causal relationship between immunophenotypes and THCA, with a sample size of about 24.2 million individuals, indicating a high statistical effect. Multiple MR analysis methods were used in this study to infer causal relationships between genetic instrumental variables, and there was no heterogeneity or horizontal pleiotropy among IVs, resulting in robust results. However, there are some limitations to this study that should be noted. First, population stratification cannot be performed because the data is not raw and specific personal data cannot be seen. Second, to obtain more IVs for horizontal pleiotropy testing and multiple sensitivity analysis, we did not use the traditional GWAS significance threshold (*p* < 5 × 10^-8^). Third, because the GWAS data for this study came from a European database, the conclusions cannot be generalized to non-European ethnic groups. Finally, since we used a more lenient threshold to evaluate the results, this may have increased some false positive rates while being able to assess the association between immune cell signatures and THCA more fully.

## 5 Conclusions

In conclusion, we explored the causal relationship between 731 immunophenotypes and THCA through MR analysis, providing new insights into the potential relationship between the immune system and THCA, providing direction and investment rationale for confirmatory experiments, and generating new hypotheses on the etiology and immunotherapy of THCA.

In addition, as long as the necessary genetic data, exposure and outcome variable data are available, it is possible to analyze the positive and negative causality of exposure and outcome without the inevitable confounders, this kind of research may carry on the low-cost, the random research to the biological pathology mechanism without outside interference. This approach can help researchers and institutions optimize the allocation of resources for expensive and resource-intensive clinical trials.

## Data availability statement

Publicly available datasets were analyzed in this study. This data can be found here: https://gwas.mrcieu.ac.uk/datasets/ebi-a-GCST90018929/.

## Ethics statement

Ethical approval was not required for the study involving humans in accordance with the local legislation and institutional requirements. Written informed consent to participate in this study was not required from the participants or the participants’ legal guardians/next of kin in accordance with the national legislation and the institutional requirements. Ethical approval was not required for the studies on animals in accordance with the local legislation and institutional requirements because only commercially available established cell lines were used.

## Author contributions

XF: Data curation, Writing – original draft. XH: Data curation, Formal analysis, Writing – original draft. JL: Data curation, Writing – original draft. DS: Writing – review & editing.
